# Hypericin Exerts Detrimental Effect on Huh-7 As a Delegacy of Hepatocellular Carcinoma: A P53 Dependent Pathway

**DOI:** 10.31661/gmj.v9i0.1896

**Published:** 2020-12-28

**Authors:** Maedeh Olya, Hamid Zaferani Arani, Amirhossein Shekarriz, Amirhossein Zabolian, Hadi Zare Marzouni, Hoda Aryan, Mohammad Hoseinian, Mohammad Amin Javidi, Hesam Adin Atashi

**Affiliations:** ^1^Young Researchers and Elite Club, Tehran Medical Sciences, Islamic Azad University, Tehran, Iran; ^2^Faculty of Pharmacy, Zanjan University of Medical Sciences, Zanjan, Iran; ^3^Student Research Committee, Mashhad University of Medical Sciences, Mashhad, Iran; ^4^Semnan University of Medical Sciences, Semnan, Iran; ^5^Department of Molecular Genetics, Faculty of Biological Sciences, Tarbiat Modares University, Tehran, Iran

**Keywords:** Hypericin, Hepatocellular Carcinoma, Huh7, P53

## Abstract

**Background:**

Hepatocellular carcinoma is the most common type of liver cancer which arises from the main cells in the liver. We address many studies investigating anti-cancer role of hypericin, however the proposing corresponding molecular pathway seems to be still a debate. Therefore, the present study aimed to evaluate the apoptotic effect of hypericin on the Huh7 as the liver cancer cell line and its relation with the gate keeper gene P53.

**Materials and Methods:**

In this study, the Huh7 cell line and fibroblast cells (as control group) were treated with different concentrations of hypericin for 24 and 48 hours. Detection of cell death was performed by MTT assay and flow cytometry. The expression of bax, bcl2 and p53 mRNAs was evaluated by Real-time PCR. Also, Immunocytochemistry (ICC) analysis was used for further evaluation of P53expression.

**Results:**

The results showed that hypericin has a dose-dependent cytotoxic effect on the Huh7 cell line, with no or marginal effect on fibroblastic cells. According to flow cytometry results, about 53%cells underwent apoptosis after exposure to LD50 of hypericin for 24 hours. Real-time PCR data demonstrated that the pro-apoptotic genes Bax and P53 expression level increased. Expectedly ICC results confirmed the up-regulation of P53 proteins in treated samples.

**Conclusion:**

Our results indicate the cytotoxicity of hypericin on Huh7 cells by affecting the expression of the gate keeper gene P53; furthermore it is suggested that this herb can be utilized simultaneously with modalities targeting P53 up-regulation or related molecular pathways.

## Introduction

Liver cancer is one of the most common types of cancer and every year, millions of people die from this disease [1,2]. Liver cancer may be caused by various factors including hepatitis C, chronic hepatitis, cirrhosis, aflatoxin fungus toxin, obesity, iron stores, excessive alcohol consumption and trisomy 18 [3-8]. The primary liver cancer begins in the liver cells and is divided into different types based on the type of cancer cell. The origin of this type of cancer can be various cells from the liver tissue, while hepatocellular carcinoma is the most common type, which originates from the main liver cells [9]. Also, due to the entrance of a large amount of blood for filtration and the metastases capability of other types of cancer in other organs, the risk of metastatic cancer in the liver increases [3,4,10,11]. Liver cancer treatment depends on the type, stage, and degree of malignancy, meanwhile is a combination of surgery, chemotherapy, and radiotherapy [12]. Surgery is a therapeutic option for people whose liver cancer is at an early stage. Usually, the surgeon removes the entire liver or part of it that is infected with cancer. If the liver is destroyed, the liver will be replaced by the liver tissue of a healthy donor. In addition, radiotherapy and chemotherapy are the next options for treating the disease. However, each of these treatments are followed by complications for the patient that may sometimes be irreparable [13,14]. Nowadays, the use of medicinal herbs has been considered in the treatment of various diseases. Investigating the diversity of plants and chemicals seems to be a vital strategy for the development of new anti-cancer drugs that are known as an alternative method with less toxicity [15,16]. The diversity of compounds derived from medicinal plants for anti-cancer and antiviral activities over the past few decades has created several drug classes that constitute a significant part of anti-cancer drug market [17]. Hypericin is the active ingredient of *Hypericum perforatum* extract. This herb has been identified by hypericin [18,19]. The therapeutic effects of hypericin have been investigated in several studies. Most of these studies have focused on an antidepressant, antiviral and antibacterial properties of hypericin [20,21]. In addition to its known antidepressant activity, hypericin appears to be a valuable source of cytotoxic compounds [22]. This results in substantial cytotoxic and pro-apoptotic effects against tumor cells that stimulate angiogenesis [23,24]. Although studies have been conducted on the anti-cancer effects of hypericin, no study has been conducted on the effect of this substance on liver cancer cells (Huh7 cell line). Therefore, the aim of this study was to evaluate the anti-cancer effect of hypericin in the treatment of liver cancer.

## Materials and Methods

###  Materials and Reagents

 The medium used to culture Huh7 cell line and normal fibroblasts (purchased from Pasteur Institute of Iran) was High glucose Dulbecco’s Modified Eagle’s Medium (DMEM) supplemented with 10% fetal bovine serum (FBS), 100 mg/ml streptomycin and 100 U/ml penicillin (all from Gibco, USA). MTT (3-[4,5-Dimethyl-2-thiazolyl]-2,5-diphenyl-2-tetrazolium bromide) assay kit which utilized to determine the biological activity of the cells and hypericin were from Sigma Aldrich (USA). To investigate whether the cellular death was a kind of apoptosis, we employed Annexin V/ propidium iodide (Annexin V/PI) apoptosis assay kit (Roche, Switzerland). Immunocytochemistry (ICC) performed, utilizing rabbit polyclonal anti-p53 antibody and goat anti-Rabbit IgG Fc (FITC), furthermore to stain cells’ nuclei we used PI (all from Abcam, UK). All other reagents were purchased from Sigma Aldrich.

###  Cell Culture

 Cells utilized in this study were cultured in the complete media described above. Cells were incubated in cell culture incubator at 37°C with 5% CO2; and changing media every 3 to 4 days. When confluency of these cells reached to 80-90%, cells were detached by trypsin. After neutralization and centrifugation, cells were counted and utilized for subsequent tests. Authenticated cells obtained from cell bank of Pasteur institute of Iran and probable contamination with mycoplasma examined by Mycoplasma PCR Detection Kit (Sigma). Experiments commenced exactly after cells were purchased, all experiments were performed with mycoplasma-free cells.

###  Cell Toxicity Measurement

We seeded 10000 and 7000 cells in each well of 96 well plates for 24 and 48 hours MTT assay, respectively. After 24 hours fresh medium with a different concentration of hypericin were replaced with the medium above each cells’ containing wells. After 24 and 48 hours, MTT assay performed as described before [25]. After formazan crystals appeared and dissolved in DMSO, absorption of each well was determined by Biotek ELX800 microplate reader at 490 nm [26].

###  Real Time Polymerase Chain Reaction (PCR)

 To perform real time PCR, total RNA was extracted from each sample treated with an appropriate dose of hypericin by TRIzol reagent (Invitrogen). The same amount of RNAs from each sample utilized for cDNA synthesis using commercial kit purchased from Takara, Japan. In this study, Q5 plex rotor gene Qiagen device (USA) was employed, and the reaction program was as follow: 1- Holding Stage: 95 °C/5 min. 2- Cycling Stage: denaturing step: 95°C/15 s, followed by annealing step 60 °C/30 s, amplification step 72°C/20s (Number of Cycles: 40). 3- Melt Curve Stage. Primers were designed to specifically amplify bax, bcl2, p53 and GAPDH (as internal control) mRNAs (Table-1).

###  ICC Assay 

 To further investigate the different expression of p53 protein in various samples, ICC test utilized. To perform ICC, cells were seeded in 12 well plates and treated with an appropriate dose of hypericin. These cells were then fixed with 4% of paraformaldehyde for 10min at room temperature. After that cells were incubated with antibody against p53 protein (from Santa Cruz biotechnology) for 16 hours at 4°C. Post this step, cells were washed with PBS and incubated with secondary anti-IgG antibody (from Santa Cruz biotechnology) containing FITC for 1 hour. PI utilized to stain cells’ nuclei and samples without primary antibody employed as a negative control.

###  Statistical Analysis

 Data was analysed by GraphPad Prism (version 5.00) using t-test (in cases which we intended to compare different groups, ANOVA performed). A P-value=0.05 was considered as the significant level.

## Results

###  MTT Assay

**Figure 1:**
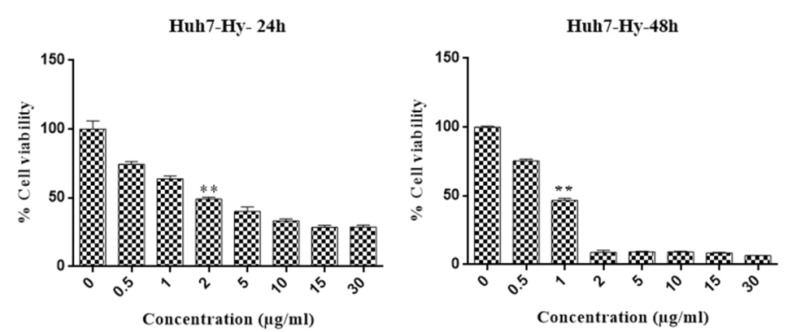
MTT assay results of the treatment of Huh7 cells with hypericin for 24 (left diagram) and 48 (right diagram) hours. As the shown, the LD50 dose estimated to be 2 μg/ml and 1 μg/ml for 24 and 48 hours after treatment, respectively.

**Figure 2:**
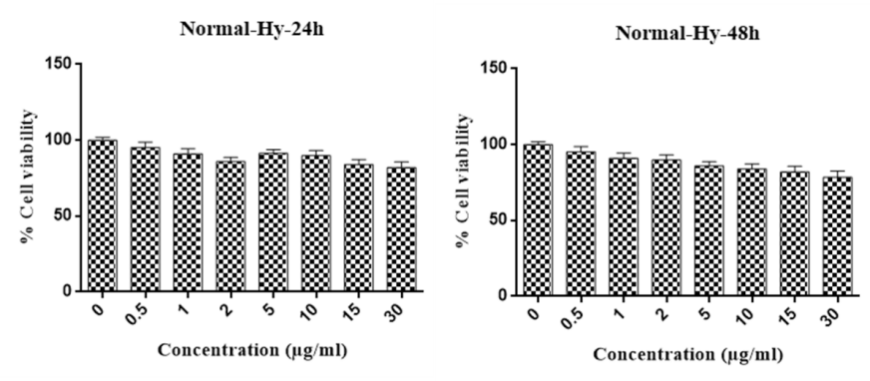
MTT assay results demonstrated that treatment of normal fibroblast cells with different doses of hypericin, the have not affected these cells viability. Left diagram, treatment of fibroblasts healthy cells with the various doses of hypericin; right diagram indicated 48 hours treatment.

 MTT results show that hypericin can induce 50% of cellular death (LD50) at the concentration of 2 μg/ml in 24 hours and 1μg/ml in 48 hours in Huh7 cells (Figure-1). Interestingly hypericin did not have a significant effect on fibroblasts cells when used concentrations up to 30 μg/ml in these times (Figure-2).

###  Flow Cytometry

**Figure 3:**
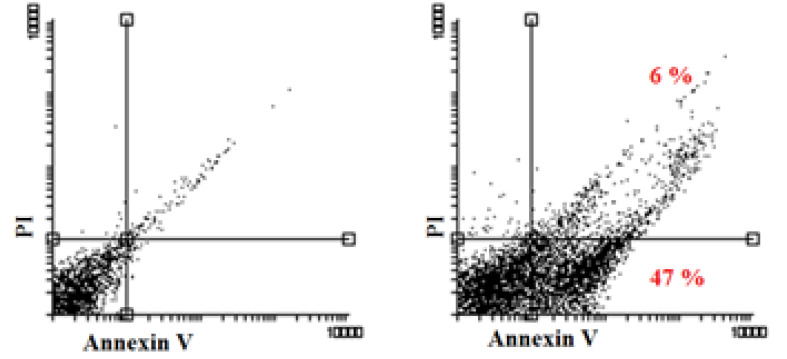
Annexin/PI apoptosis assay. Treatment of Huh7 cells with an LD50 dose of hypericin for 24 hours, induced 53% early and/or late apoptosis in these cells (left diagram; untreated Huh7 cells, right diagram; Huh7 cells which treated with LD50 dose of Hypericin for 24 hours).

Annexin V/PI test demonstrated that cellular death induced by hypericin was apoptosis. As it is shown in figure 3, treatment of Huh7 cells lines with a LD50 dose of hypericin for 24 hours, induced 53% apoptosis (Figure-3).

###  Expression Level of Apoptotic by mRNAs and ICC 

**Figure 4:**
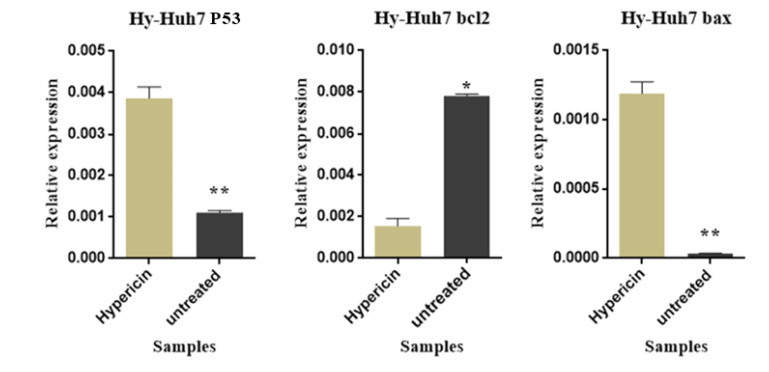
Real time PCR results confirmed that treatment of Huh7 cells causes apoptotic genes overexpression (p53 and bax; right). Furthermore, this treatment down regulates the expression levels of antiapoptotic gene bcl2.

**Figure 5:**
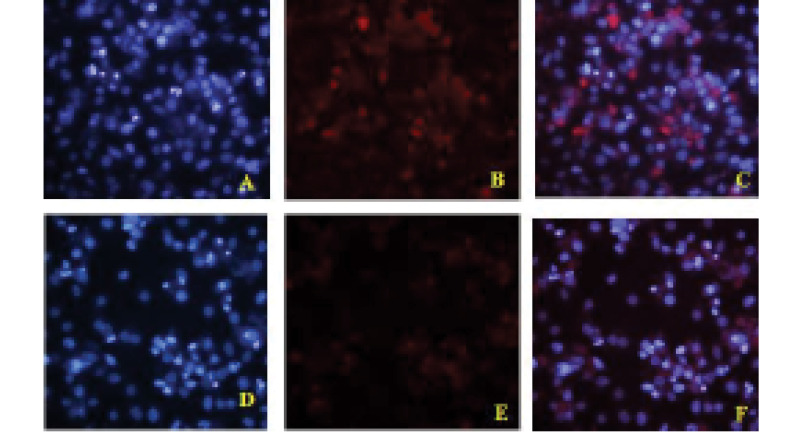
ICC test results. Treatment of Huh7 cells with LD50 dose of hypericin for 24 hours, causes the overexpression of p53 proapoptotic proteins (A-C in this figure) compared to untreated cells (D-F). A and D; Huh7 cells’ nuclei which stained with DAPI. B and E staining of cells with an antibody against p53 proteins, secondary antibody, and FITC. C and F merge picture of either A+B or D+E. Red dots show the expression of p53 proteins.

 To further confirm that hypericin induces apoptosis in Huh7 cells, mRNA expression level of bax, bcl2, and p53 were investigated. Post treatment by LD50 dose of hypericin for 24 hours, bcl2 mRNA expression level decreased, and p53 and bax mRNA expression levels increased significantly (Figure-4). By performing ICC, we further confirmed that treatment of cells with 24 hours LD50 of Hypericin induces the expression level of P53 t protein level. Cells which treated by LD50 dose of hypericin expressed p53 protein much more than untreated cells (Figure-5).

## Discussion

Liver cancer can lead to irreparable complications due to the vital role of this organ in the body. Treatment of liver cancer is done through surgery, radiotherapy, and chemotherapy. Considering that resistance to radiotherapy and chemotherapy is always a common problem, the identification, and development of anti-cancer drugs is always a vital requirement [25]. *H. perforatum* is a member of Hypericaceae family, and its active ingredient is hypericin. Some studies have reported the benefits of hypericin in the treatment of some of the cancer cases [19,22]. However, the mechanism of hypericin in treating cancer cells is unclear, but it is believed that this active ingredient affects a number of essential cell lines from different cancers including breast cancer. The results of this study demonstrated that hypericin has significant cytotoxic effects on Huh7 cancer cells as a representative of hepatocellular carcinoma, the most type of liver cancer. LD50 for this extract was 2μg/ml, 24 hours after exposure, and it was 1μg / ml, 48 hours after exposure. One important point in the results of this study is that the results showed no effect on fibroblast cells even at high doses. This indicates that hypericin has no effects on healthy cells. In a study by Hamilton *et al*. they confirmed that at low concentrations, hypericin inhibits the growth of pituitary adenoma cells in AtT-20 and GH4C1 cell lines. Also, the effect of this substance on fibroblast cells was confirmed [27]. Kim *et al*. demonstrated that growth inhibitor concentration in 50% of U-937 cells associated with myeloid leukemia is 0.2 µM [28]. Moreover, in a study by Mirmalek *et al*. on the cytotoxic and apoptotic effect of hypericin on MCF-7 cell line, they concluded that the hypericin plays a dose-dependent cytotoxic effect on this cell line. They showed that the LD50 of cisplatin vs. hypericin on the MCF-7 cell line was 5 μg / ml vs. 20 μg / ml, indicating the effectiveness of this substance at much lower concentrations compared with cisplatin. In their study, they concluded that hypericin has an appropriate cytotoxic effect on the MCF-7 cell line and is a suitable candidate to be used in the treatment of this type of cancer [22]. In our study, we used flow cytometry and ICC to determine the level of apoptosis and cytotoxicity induced by exposure to hypericin, and real-time PCR was used to measure the expression levels of bax, bcl2, and p53 mRNAs. Our results indicate 53% apoptosis induced by 24 hours exposure of 2 μg/ml hypericin to the Huh7 cell line. In the study of Mirmalek *et al*. the apoptosis induction rate at a concentration of 5 μg/ml on MCF-7 cell lines was 52% [22]. The ICC results of the present study confirmed these findings. In addition, the current study showed that the expression levels of p53 and bax mRNA was increased after exposure to hypericin for 24 hours and the expression level of bcl2 mRNAs decreased. These results confirm the induction of apoptosis and cytotoxic activity of hypericin on the studied cell line. These results are consistent with the studies of Mirmalek *et al*. and Acar *et al*. [22,29]. Acar *et al*. indicated that hypericin induction increases expression of cell death inducing genes and, ultimately, increase apoptosis. Some studies have examined the mechanism of action of hypericin in the induction of cancer cell apoptosis and its cytotoxic effects. For example, in the study of Jendzelovsky *et al*., it was argued that hypericin exposure could increase expression in the multidrug resistance-related protein 1 (MRP1) and breast cancer resistance protein (BCRP) genes [30]. The expression of these two genes can be related to the linkage between proadifen (SKF525A) and ABC transporter proteins. This relationship increases intracellular oxidative stress and decreases the mitochondrial membrane potential, which is associated with the activation of caspase 9 and then caspase 3, and ultimately leads to apoptosis. In a study by Eriksson *et al*., it is argued that there is the possibility of interactions between hypericin and Ca2+^2+^pump SERCA in terms of the 3D structure [31]. They also managed to show the presence of a proper location of the cell membrane for the binding of hypericin to some parts of the membrane lipid. An important part of these location exists in the endoplasmic membrane. In addition, in a study by Barliya *et al*., it was indicated that hypericin plays a key role in the induction of hypoxia-inducible factor 1α, thereby eliminating von-Hippel Lindau protein from cancerous cells and preventing the growth of cancer cells [32]. The results of our study demonstrated that hypericin inhibits cell division and apoptosis in these cells, while several studies confirm the results of this study [22,33].

## Conclusion

Our results study indicated that hypericin is a cytotoxic and apoptosis-inducing substance in Huh7 cell line. However, this substance does not have significant cytotoxic effects on fibroblast cells at the time/dose utilized for treatment in this study. Considering the endogenous overexpression of P53 occurred post hypericin treatment; it is suggested that this herb can be utilized simultaneously with modalities targeting P53 up-regulation or related molecular pathways. It seems that this substance, with its proper anti-cancer effects, can be a suitable alternative for the treatment of this disease. It is suggested that in future studies, the anti-cancer effects of this substance be evaluated under *in vivo* conditions.

## Acknowledgment

 The authors intend to thank Prof. Majid Sadeghizadeh and Dr. Ehsan Janzamin for their collaboration in this study.

## Conflict of Interest

 The authors declare that they have no competing interests.

**Table 1:** Primers Sequences Used to Amplify Specifically Bax, bcl2 and p53 mRNAs.

**Gene**	**Primers sequences**	**Amplicon**
**Bax**	F:GCAAACTGGTGCTCAAGG R:CAGCCACAAAGATGGTCA	183 bp
**Bcl2**	F:CTGCACCTGACGCCCTTCACC R:CACATGACCCCACCGAACTCAAAGA	119 bp
**P53**	F:TCCTCAGCATCTTATCCGAGTG R:AGGACAGGCACAAACACGCACC	265 bp
